# Revisiting the *Cellulosimicrobium cellulans *yeast-lytic β-1,3-glucanases toolbox: A review

**DOI:** 10.1186/1475-2859-5-10

**Published:** 2006-03-17

**Authors:** Pau Ferrer

**Affiliations:** 1 Department of Chemical Engineering, Universitat Autònoma de Barcelona, 08193- Bellaterra (Cerdanyola del Vallès), Spain

## Abstract

*Cellulosimicrobium cellulans *(also known with the synonyms *Cellulomonas cellulans, Oerskovia xanthineolytica*, and *Arthrobacter luteus*) is an actinomycete that excretes yeast cell wall lytic enzyme complexes containing endo-β-1,3-glucanases [EC 3.2.1.39 and 3.2.1.6] as key constituents. Three genes encoding endo-β-1,3-glucanases from two *C. cellulans *strains have been cloned and characterised over the past years. The *βglII *and *βglII*_*A *_genes from strain DSM 10297 (also known as *O. xanthineolytica *LL G109) encoded proteins of 40.8 and 28.6 kDa, respectively, whereas the β-1,3-glucanase gene from strain ATCC 21606 (also known as *A. luteus *73–14) encoded a 54.5 kDa protein. Alignment of their deduced amino acid sequences reveal that βglII and βglII_*A *_have catalytic domains assigned to family 16 of glycosyl hydrolases, whereas the catalytic domain from the 54.5 kDa glucanase belongs to family 64. Notably, both βglII and the 54.5 kDa β-1,3-glucanase are multidomain proteins, having a lectin-like C-terminal domain that has been assigned to family 13 of carbohydrate binding modules, and that confers to β-1,3-glucanases the ability to lyse viable yeast cells. Furthermore, βglII may also undergo posttranslational proteolytic processing of its C-terminal domain, resulting in a truncated enzyme retaining its glucanase activity but with very low yeast-lytic activity. In this review, the diversity in terms of structural and functional characteristics of the *C. cellulans *β-1,3-glucanases has been compiled and compared.

## Review

Several bacteria have been reported to be able to lyse and grow on viable yeast and fungal cells by producing a variety of cell-wall degrading enzymes such as endo-β-1,3-glucanases, proteases, β-1,6-glucanases, mannanases, and chitinases. The structural complexity of the yeast cell wall, which is mainly composed of complex polymers of β-1,3- and β-1,6-glucans, mannoproteins, and smaller amounts of chitin [1,2], implies that the synergistic action of these enzymes is necessary to hydrolyse its components into assimilable substrates. Nevertheless, endo-β-1,3-glucanases [EC 3.2.1.39 and EC 3.2.1.6] have been considered to play a major role in yeast cell lysis [2,3].

The actinomycete *Cellulosimicrobium cellulans *(also known with the synonyms *Cellulomonas cellulans, Oerskovia xanthineolytica*, and *Arthrobacter luteus*), has been regarded as a major source of yeast-lytic enzymes, particularly endo-β-1,3-glucanases, proteases and mannanases. Notably, several commercially available yeast-lytic glucanases preparations derived from this organism, namely Lyticase, Zymolyase, and Quantazyme, have been widely used for yeast protoplast preparation and yeast DNA isolation. Endo-β-1,3-glucanases are the major component of such enzyme preparations. Only one of these commercially available preparations (Quantazyme, Quantum Biotechnology, Canada) is produced recombinantly and protease-free. Besides their application in spheroplasting, *C. cellulans *β-1,3-glucanases have shown their big potential in a wide range of applications in both basic research and biotechnology; for instance, in structural analyses of the yeast and fungal cell wall [4,5], in cell wall permeabilisation for the selective recombinant protein recovery from yeast cells [6,7], or in biocatalysis [8-10], among others.

Early characterisation studies on the lytic enzyme system from different *C. cellulans *strains showed that this organism excreted a wide heterogeneity of β-1,3-glucanase forms with different physicochemical and functional properties [11-15]. Notably, while all of the isolated forms showed hydrolytic activity toward β-glucans (glucanase activity), only some were found capable of inducing lysis of viable yeast cells (lytic activity).

The cloning and sequencing of three β-1,3-glucanase-encoding genes from *C. cellulans *has allowed for further molecular and biochemical characterisation studies over the past years. These studies have revealed the diversity in terms of structural and functional characteristics of the *C. cellulans *β-1,3-glucanases, which have been compiled and compared in this review.

### Origin of the multiplicity of β-1,3-glucanases isoforms

Over the past years, three genes encoding β-1,3-glucanases from two different *C. cellulans *strains (ATCC 21606 and DSM 10297) have been cloned and characterised (table 1), [16-20]. The *βglII *and *βglII*_*A *_genes from strain DSM 10297 (formerly *O. xanthineolytica *LL-G109) encoded mature proteins of 40.8 and 28.6 kDa, respectively, whereas the β-1,3-glucanase gene from strain ATCC 21606 (formerly *Arthrobacter luteus *73–14) encoded a 54.5 kDa protein. These studies revealed that the multiplicity of β-1,3-glucanase forms excreted by this organism may be the result of the coexistence of multiple glucanase-encoding genes. In addition, there is strong evidence that proteolytic processing of some of these enzymes may also generate derivatives retaining β-1,3-glucanase activity. In particular, the single molecular specie of the major β-1,3-glucanase activity initially purified and characterised from *C. cellulans *DSM 10297 cells growing on yeast glucan, had a molecular weight of about 27.2 kDa, as determined by mass spectrometry [21], and a pI of 4.85~5.0 [21]. However, the mature βglII deduced sequence is a 383 aa polypeptide with a predicted molecular weight of 40,8 kDa and pI of 5.73 (as calculated with the sequence analysis tools at ), comprising a larger N-terminal catalytic domain and a C-terminal carbohydrate-binding domain of about 120 aa, as discussed below. This suggested that the 27.2 kDa enzyme actually corresponded to a C-terminal-truncated form of βglII (βglIIt) generated after its post-secretional proteolytic processing. This was corroborated by the fact that i) the predicted 27.2 kDa proteolytic product derived from the 40.8 kDa βglII, which would correspond to a polypeptide of about 250 residues and a calculated pI of 5.37, had a deduced amino acid composition in concordance with that experimentally determined for the purified native β-1,3-glucanase [22]; ii) partial amino acid sequences of the purified native β-1,3-glucanase were homologous to corresponding N-terminal and internal βglII sequences [17]; iii) the purified native 27.2 kDa β-1,3-glucanase had higher affinity towards soluble β-1,3-glucan (laminarin) than insoluble yeast β-glucan (and, therefore, very low yeast-lytic activity) [22], and iv) heterologous expression of βglII in *Escherichia coli *yielded two molecular forms: a 40- and a 27.2-kDa protein having β-1,3-glucanase activity, with a experimentally determined pI of 6.3 and 4.8, respectively [20].

**Table 1:** Summary of β-1,3-glucanases from *Cellulosimicrobium cellulans*

**Enzyme**	**Catalytic domain GH family**	**MW (kDa)**	**pI**	**K_m _(mg ml- ^1^) ^*c*^**		**pH optimum **^*c*^			**Temp. optimum**	**Lytic activity^*b*^**	**Reference**
				laminarin^*a*^	yeast glucan	laminarin	yeast glucan	yeast cells			

***Strain DSM 10297 ***Native βglIIt	16	27.2	4.85~5.0	0.95	2.5	6.0	8.0		65	very low	21, 22
rβglII	16	40.8	6.3	2.75		8^*d*^			40		20
rβglIIt	16	27~30^*e*^	4.8								20
rβglII_*A*_	16	28.6	3.8~4.0			~4.0					18
											
***Strain TK-1***											
Native β-1,3- glucanase	16	40	6.5			7.5	5.5		60	high	26
											
***Strain ATCC 21606***											
Native β-1,3- glucanase	64	54.5		5.9	0.4	6.0		7.5~8.0^*d*^		high^*m*^	15
rβ-1,3- glucanase	64	54.5								high	16
rβ-1,3- glucanase(t)	64	42	6.1							absent	16

Strain ATCC21606 is also known as *A. luteus *73/14

Strain DSM10297 is also known as *O. xanthineolytica *LL-G109

^*a *^Assuming a molecular weight of 4000 for laminarin

^*b *^Using *S. cerevisiae *cells as substrate

^*c *^at 30°C

^*d *^at 37°C

^*e *^estimated from SDS-PAGE analysis

^*m *^needs 2-mercaptoethanol

r recombinant form

t truncated form

In contrast, only one gene could be isolated from a *C. cellulans *ATCC 21606 genomic library [16]. Nevertheless, southern blot hybridization studies suggest that strain ATCC 21606 also has a βglII-like gene [23].

### Functional properties of *C. cellulans *β-1,3-glucanases

As discussed below, while the β-1,3-glucanase isolated from strain ATCC 21606 has been classified in family 64 of glycosyl hydrolases (GH-64), β-1,3-glucanases from strain DSM 10297 have been classified in family 16 of glycosyl hydrolases (GH-16), thus revealing an important structural diversity [24,25]. This is further reflected in the heterogeneity of *C. cellulans *functional characteristics:

Although all *C. cellulans *β-1,3-glucanases hydrolyse yeast glucan in an endolytic manner, GH-16 β-1,3-glucanases yield a mixture of biose and glucose [26], whereas GH-64 β-1,3-glucanases hydrolyse yeast glucan with predominant liberation of pentoses [15].

Enzymatic hydrolysis of glycosidic bonds occurs with two possible stereochemical outcomes: inversion or retention of the anomeric configuration at the site of cleavage. 'Inverting' enzymes utilise a single-displacement reaction where an activated water molecule performs a nucleophilic attack at the sugar C-1 while concomitant aglycone departure is achieved by protonation of the glycosilic oxygen. By contrast, 'retaining' enzymes utilise a double-displacement mechanism involving a covalent glycosyl-enzyme intermediate.

The stereochemistry of hydrolysis in family GH-64 β-1,3-glucanases has been recently determined for one of its members, a β-1,3-glucanase from *Streptomyces matensis *[27]. Interestingly, while family GH-16 β-glucanases have been shown to be 'retaining' enzymes [28], this GH-64 enzyme is the first inverting β-1,3-glucanase characterised. The inverting mechanism implies that the molecular mechanism of hydrolysis by this enzyme does not involve the formation of a covalent glycosyl-enzyme intermediate. Since the molecular mechanism has been shown to be conserved within the families of glycoside hydrolases, it can be concluded that family GH-64 glycoside hydrolases may operate by an inverting mechanism [27].

The differences in "yeast-lytic" activity of the different β-1,3-glucanases forms is reflected in their kinetic properties (table 1). For instance, the 27.2 kDa βglIIt form (i.e. with no carbohydrate binding module) has very low yeast lytic activity; correspondingly, its *K*_*m *_for yeast glucan (insoluble) is higher than for the soluble substrate (laminarin). In contrast, a β-1,3-glucanase with high lytic activity such as the one from ATCC 21606 strain (having a C-terminal carbohydrate-binding domain, as discussed below), has a *K*_*m *_for yeast glucan lower than for laminarin [16]. Another interesting observation concerns the possible effect of a carbohydrate-binding domain in the catalytic properties for soluble substrates of the catalytic domain is attached to. In particular, the presence of a carbohydrate-binding domain in the recombinant βglII seems to increase the *K*_*m *_for laminarin in relation to the native βglIIt form (table 1), [20].

β-1,3-glucanase forms isolated from *C. cellulans *appear to have a pH optimum in the range of 5.5 to 8 (depending on the substrate), with the exception of the βglII_*A *_enzyme from strain DSM 10297, which seems to have an acidic pH of about 4.0 [18]. It is also remarkable that the GH-16 β-1,3-glucanases from *C. cellulans *so far characterised (βglII from strain DSM 10297 and the β-1,3-glucanase from the strain known as *O. xanthineolytica *TK-1 [26]) have a moderately high optimum activity temperature (table 1). However, only the native βglIIt enzyme has been shown to have a significant thermotolerance (the enzyme retained about 50% of its residual activity after 30' of incubation at 70°C, pH 7 [21]).

### Sequence analysis of *C. cellulans *β-1,3-glucanases

**Figure 1:**
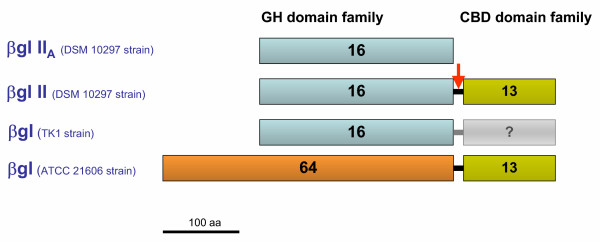
**Structural organisation of the *C. cellulans *β-1,3-glucanases. **Family 16 catalytic domain (blue boxes), family 64 catalytic domains and CBM13 domains (green boxes) are depicted for each enzyme. The β-1,3-glucanase from strain TK-1 has not been fully sequenced and, therefore, its putative CBM domain (if present) has not been assigned. The vertical arrow indicates the proteolytic cleavage of βglII yielding its catalytically active truncated form.

Similarity searches using BLAST [29] at the National Center for Biotechnology Information server [30], showed that βglII is related to the GH-16 family, (see the CAZy database, [31-33]). Sequence alignments with proteins of this family suggest that βglII comprises a catalytic domain of ~240 aa, which is connected by a small glycine-, serine- threonine- and proline-rich 23 aa-linker region to a 120-aa C-terminal domain consisting of a tandem of three imperfect repeats, R1, R2, and R3. This domain shows high sequence similarities to a number of Carbohydrate-Binding Modules (CBMs) found in diverse glycosyl hydrolases, as well as to several plant lectins (see updated CBMs at the CAZy database, [31-33]). In contrast, βglII_A _is a non-modular enzyme, i.e. it only has a catalytic domain [18] (figure 1). The βglII catalytic domain is highly similar to βglII_*A *_(80.3% sequence identity over a 245 aa overlap). Interestingly, both isoenzymes seem to have different catalytic properties, e.g. different optimum pH of activity (table 1). Also, βglII has homologous N-terminal amino acid sequence (over the first 33 residues), as well as similar molecular weight and pI, to the yeast-lytic β-1,3-glucanase purified from the TK-1 strain (table 1) [26].

In contrast, the 380 aa catalytic domain of the yeast-lytic 54.5 kDa β-1,3-glucanase from *C. cellulans *ATCC 21606 [16], has been classified into GH-64 family. Interestingly, this β-1,3-glucanase is a modular enzyme, having a C-terminal "lytic domain" of about 120 aa that falls into the CBM family 13 (figure 1).

βglII shows the highest similarity values, ranging from about 60 % to 40 % of sequence identity in 240 aa overlaps, to the GH-16 bacterial endo-1,3-β-glucanases (laminarinases) subfamily members, as well as to several non-bacterial β-1,3-glucan-acting proteins such as the β-1,3-glucanase LamA from the archeon *Pyrococcus furiosus *(GenBank accession number AF013169) [34], 49 % identity, the β-1,3-glucanase from *Strongylocentrotus purpuratus *(sea urchin), (GenBank accession number U49711) [35], 36.6% identity, and to the α-subunit of the (1→3)β-D-glucan-sensitive coagulation factor G from *Tachypleus tridentatus *(horseshoe crab), (GenBank accession number D16622) [36], 39.5% identity. Sequence identity values between βglII and the GH-16 bacterial 1,3-1,4-β-glucan 4-glucanohydrolases (lichenases) subfamily members are somewhat lower (ca. 25%). Detailed similarity and phylogenetic analyses have been reported for GH-16 enzymes [28,37]. Secondary structure analyses of the native 27.2 kDa βglIIt form revealed a high content of β-structure and the presence of a compact hydrophobic core including the presence of several tryptophan residues [21], which is consistent with the characteristic jellyroll β-sandwich fold of bacterial family 16 β-glucanases [28].

The sequence WPSSGEIDIME, which includes de catalytic glutamate residues of the active site conserved within GH-16 [38], was identified between residues 166 to 176 and 177 to 187 of the βglII and βglII_*A *_precursors, respectively. Also, the Met residue of this motif, which is invariant in GH-16 laminarinases subfamily but not present in the active site of the GH-16 lichenases subfamily members, is likely to have an important structural role in the active site of βglII, as observed in the *Rhodothermus marinus *LamR laminarinase and bglA β-glucanase [39,39] and the archeon *Pyrococcus furiosus *LamA laminarisase [34]). Notably, the βglIIt form purified from strain DSM 10297 has been shown to be able to hydrolyse both β-1,3- and β-1,3-1- 4-glucan (lichenan) [21], as reported for some other members of the GH-16 laminarinases subfamily [39,39]. As noted earlier [18], it is remarkable that GH-16 β-1,3-glucanases have 9 highly conserved tryptophan residues.

Sequence identity of the 54.5 kDa β-1,3-glucanase from *C. cellulans *ATCC 21606 GH-64 catalytic domain with other β-1,3-glucanases of this family of glycosyl hydrolases ranges from 99% identity to the β-1,3-glucanase of *Arthrobacter *sp. YCWD3 (GenBank accession number D23668) to 60% and 31% to the Laminaripentaose-Producing β-1,3-glucanase of *Streptomyces matensis *DIC-108 [40] and the β-1,3-glucanase B from *Lysobacter enzymogenes *[37], respectively. In contrast to β-1,3-glucanases from strain ATCC 21606 and *Arthrobacter *sp. YCWD3, the β-1,3-glucanases from *S. matensis *and *L. enzymogenes *do not contain the carbohydrate-binding modules at the C-terminus. This indicates that the liberation of only laminaripentaose as the degradation product from β-1,3-glucan observed for this family of enzymes is not related to their CBM. The 54.5 kDa β-1,3-glucanase GH-64 catalytic domain from strain ATCC 21606 shares a very low sequence identity to βglII and βglII_*A *_GH-16 catalytic domains (15% and 19%, respectively, over the entire domain). Similarity and phylogenetic analyses have also been reported for GH-64 enzymes [37].

**Figure 2:**
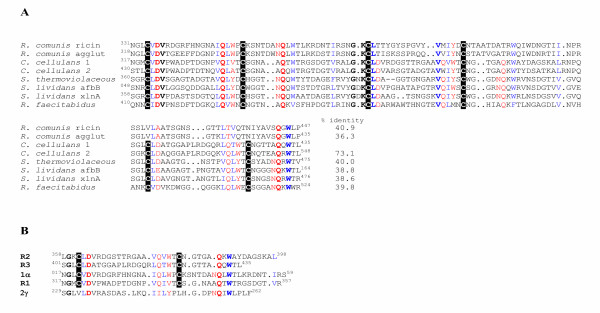
**Amino acid sequence alignments of family 13 carbohydrate binding modules. **Sequences were deduced from the following GenBank accession numbers: *C. cellulans *DSM 10297 βglII glucanase (AF052745) [19] (indicated as *C. cellulans *1); *C. cellulans *ATCC 21606 β-1,3-glucanase (M60826) [16] (indicated as *C. cellulans *2), *Ricinus comunis *agglutinin β-chain (M12089) [42], *Ricinus comunis *ricin β-chain (X52908) [43], *Streptomyces thermoviolaceus *xylanase I (AB110643) [49], *Streptomyces lividans *α-arabinofuranosidase and xylanase A (M64551) [51], and *Rarobacter faecitabidus *Protease I (D10753) [41]. Comparison of the βglII CBM amino acid sequence with related CBMs. The amino acid residues conserved in all of the sequences (boldface), the residues involved in galactose binding in the ricin β-chain subdomains α 1 and γ2, and the corresponding analogous positions in all the subdomains of the aligned sequences (in red), conserved residues that form the hydrophobic core of each ricin β-chain subdomain (in blue), and conserved Cys residues (black background) are marked. Sequence identity values (in %) are referred to the *C. cellulans *βglII sequence. (A) Sequence similarity of the R1, R2, and R3 subdomains of the βglII CBM, and the subdomains α 1 and γ2 of the ricin β-chain.

The sequence identity between each of the three imperfect repeats found at the C-terminal region of βglII ranges from 53.3% to 36.8%. A similar set of repeats are found in the C-terminal CBM of the *C. cellulans *ATCC 21606 β-1,3-glucanase, as well as in other yeast-lytic enzymes, namely in the C-terminal mannose-binding domain of the *Rarobacter faecitabidus *Protease I (D10753) [40]. As mentioned, all these domains belong to the CBM family 13. The members of this CBM family usually have approximately 150 residues, which always appear as a threefold internal repeat. These modules were first identified in several plant lectins such as ricin or agglutinin of *Ricinus communis*, which bind galactose residues. Family 13 CBMs are also found in bacterial proteins with rather diverse functions, e.g. xylanases, arabinofuranosidases, β-1,3-glucanases, and proteases (CAZy database, [31-33]). Moreover, it also includes the central domain of the subunit α of the horse-shoe crab (1→3)-β-D-glucan sensitive coagulation factor precursor (GenBank accession number D16622), [36]. Alignment of these sequences reveals the presence of 6 very well conserved cysteine residues, two per repeat (Figure 2a). Besides, the C-terminal repeats from βglII and the β-1,3-glucanase from the ATCC 21606 strain have significant similarities with the repeats found in several plant lectins such as the galactose-binding specific rRNA N-glycosilases (ricins and agglutinins) from *Ricinus comunis *(castor bean) (GenBank accession numbers M12089 and X52908), [43,43]. Pair wise similarities of these protein domains with the 120-aa C-terminal region of βglII range between 73.3% and 36.3% of identity in 120 aa overlaps (Figure 2a). Although knowledge about the structure-function relationships of the family 13 CBM domains is limited, important information can be derived from the X-ray and site directed mutagenesis studies of the ricin β-chain [44]. This lectin chain is composed of two major domains, 1 and 2, each of which has a galactose-binding site. Each of these domains contains three copies (α, β, and γ) of a primitive 40-residue folding unit or subdomain. Subdomains α_1 _and γ_2 _contain the galactose-binding sites. A detailed local similarity analysis (Figure 2b) of the C-terminal region of the βglII precursor (^317^Asn – ^435^Leu) reveals that the 40 aa-repeats (R1, R2, and R3) from this enzyme show significant sequence identities with repeats α and β of the ricin β-chain (e.g. 45% identities between R1 and α_1_). Similarities of R1, R2, and R3 to γ subdomains are lower (e.g. 30% identities between R1 and γ_2_). Since cysteine positions in subdomains α and β are conserved and known to form disulfide bonds, cysteine residues in each of the βglII repeats may possibly be involved in disulfide bond formation (i.e. ^320^Cys-^339^Cys, ^361^Cys-^378^Cys, ^405^Cys-^423^Cys), and play an important role in the tertiary structure of this region of the protein. In addition, residues ^22^Asp, ^35^Gln, ^37^Trp, ^46^Asn, and ^47^Gln in the α_1 _subdomain from the ricin β-chain, which are known to form the galactose-binding pocket, are also conserved (except ^46^Asn) in each of the three repeats of βglII (Figure 2b). Furthermore, the hydrophobic residues ^21^Val, ^34^Ile, ^36^Leu, ^49^Trp, and ^57^Ile in the α_1 _subdomain, where they play an important structural role in the preservation of its tertiary structure, are also conserved in subdomains R1, R2, and R3. Overall, one can predict that each repeat in the C-terminal region of βglII may constitute a subdomain with a similar fold to the α, β, and γ subdomains of the ricin β-chain.

Truncated yeast/fungi-lytic β-glucanases, chitinases and proteases lacking their corresponding CBMs show reduced activities against viable yeast/fungal cells while retaining their capacity to depolymerise colloidal glucan or chitin, or to degrade proteins [16,41,46,46]. As summarised above, the proteolytic removal of the CBM from βglII dramatically reduces its capability to lyse viable yeast cells. However, the ability of the βglIIt form to lyse viable yeast cell walls is restored in the presence of the yeast-lytic protease component secreted by *C. cellulans *[22]. This synergistic effect between the βglIIt form and the lytic proteases suggests that the affinity of this β-1,3-glucanase for the glucan layer of the cell wall does not depend on the possession of the carbohydrate-binding domain, as it can readily solubilise the glucan component of the yeast cell wall when the outer mannoprotein layer is removed by the proteases. However, there is no reported evidence on whether the CBM may have any effect on the *K*_*m *_values of βglII for insoluble substrates such as yeast glucan. Affinity of the βglII catalytic domain to polysaccharides could be partially conferred by some of the highly conserved tryptophan residues, as observed in some polysaccharide-binding proteins [47]. Interestingly, some of these residues are believed to be located at the surface of the GH-16 LamR laminarinase from *R. marinus *[38].

Considering that the ricin B-chain exhibits galactose-binding activity and has a specifically high affinity for the oligosaccharides from cell wall surfaces (it binds much more strongly to complex galactosides from cell wall surface carbohydrates than to simple sugars, [48]), its similarity to the βglII C-terminal repeats suggests that these constitute a lectin-like domain with binding activity towards oligosaccharides of the yeast cell wall surface, which are rich in mannose. In addition, the similarity of this domain with the *R. faecitabidus *yeast-lytic Protease I mannose-binding domain and the strain ATCC 21606 β-1,3-glucanase carbohydrate-binding domains, leads to the conclusion that the C-terminal domain of these *C. cellulans *β-1,3-glucanases is a mannose-binding module, and that it is also essential for efficient lytic activity towards viable yeast cells. Mannose-binding domains may play an important general function in targeting yeast/fungi-lytic enzymes to their substrates by increasing their local concentration on the yeast/fungal cell wall surface, which is rich in mannoproteins. Recent studies on the *S. cerevisiae *cell wall architecture using Quantazyme *ylg *(i.e. the pure recombinant β-1,3-glucanase preparation from *C. cellulans *ATCC 21606) have revealed that this enzyme is able to release cell wall mannoproteins by cleaving β-1,3-chains, to which these cell wall proteins are attached [4]. However, the mode of action of βglII and its precise target on its natural substrate, the yeast cell wall, is still unknown. It is significant that *C. cellulans *can co-produce modular and non-modular β-1,3-glucanases, either by proteolytic digestion of modular species, or by expressing specific genes, suggesting that these truncated versions have also an important role in cell wall degradation (figure 1). At this stage, it is apparent that more comprehensive studies are needed in order to evaluate the specific role of modular and non-modular yeast/fungi lytic β-1,3-glucanases on the yeast cell wall degradation, and their interactions with other lytic enzymes secreted by these bacteria.

Besides *C. cellulans*, there are other prokaryotes, such as *L. enzymogenes *and *S. coelicolor*, known to produce multiple β-1,3-glucanase systems with the ability to lyse fungal/yeast cells. These three species contain both GH-64 and GH-16 enzymes. Furthermore, these β-1,3-glucanase systems share significant similarities in terms of structural organisation. For instance, GluC and GluA from *L. enzymogenes*, and βglII and βglII_*A *_from *C. cellulans *have GH-16 catalytic domains;gluC and βglII contain a substrate-binding domain located at their C-terminal that is lacking in GluA and βglII_*A*_. Interestingly, the substrate-binding C-terminal regions observed in some of these β-1,3-glucanases belong to different CBM families, namely family 13 for *C. cellulans *glucanases and, family 6 for *L. enzymogenes *[37]. This diversity observed among enzyme type and source organism is a trait indicative of domain shuffling in the evolution of glycosyl hydrolases.

## Conclusion

Availability of recombinant *C. cellulans *β-1,3-glucanases has opened the door to comprehensive characterisation (and future engineering) of these biotechnologically important enzymes, which is key for the development of new/potential applications or the optimisation the existing ones. Nevertheless, a better understanding of the basis of the substrate specificity and interactions with the yeast cell wall components still awaits a detailed comparison of the three-dimensional structures of these enzymes and systematic experimental verifications of the derived conclusions by protein engineering.
